# The inclusion of Gambling and other behavioral addictions in the addictions chapter of ICD-11

**DOI:** 10.1192/j.eurpsy.2025.74

**Published:** 2025-08-26

**Authors:** S. Achab

**Affiliations:** University of Geneva, Geneva, Switzerland

## Abstract

**Abstract:**

**Background and relevance**

Addictive behaviours have for the first time been included within the addiction spectrum. The transition to ICD-11 is a critical step in advancing mental health and addiction medicine.

The implementation of ICD-11 is expected to improve Health Records, facilitate Better Treatment, reduce Stigma, and enhance Public Health Surveillance.Testing the practical implications of these changes is key to ensure preparedness and smooth and effective implementation.

**Methods:**

We conducted a comprehensive evaluation to assess the practical implications of these changes in Switzerland, determining the ease of implementation in clinical and public health settings.

Assessment consisted in:

Gathering Feedback through collecting insights from health professionals to identify potential challenges and areas for improvement.

Informing Training Needs by identifying the training requirements for health professionals to effectively use the new system.

The field testing involved multiple components: online survey, focus groups, expert reviews, key informant interviews, and a consensus conference.

**Results:**

The testinginvolved 64 health professionals and covered diverse professional backgrounds and geographical locations). Respondents had a median of 15.25 years of experience in addiction medicine, 75% of them were Clinicians and 25% were Public Health Professionals.

Key findings of the diffrernt survey components included: High approval rates for the utility (89%), feasibility (92%) and ease of use (86%) of the ICD-11.

Strong support (90%) for the inclusion of Addictive behaviors, particularly Gaming disorder and Gambling disorder, In contrast, the broader clinical spectrum of Gaming disorder (hazardous gaming and harmful gaming) received limited support (5%).

The national consensus conference concluded that ICD-11 is mainly perceived to have a positive impact on Swiss health records, to enable better documentation of emerging health conditions, and to reduce stigma. Divergent views were represented and expressed their acceptance or reluctance towards including addictive behaviours.

Careful transition and preparedness at the national level, training health professionals along with effective communication to public audience, were reported to be essential to mitigate risks of stigma and over-pathologizing addictive behaviours.

**Conclusion:**

The implementation of ICD-11 in Switzerland is anticipated to enhance the understanding of the societal and economic impacts of new categories such as Gaming disorder. The field-testing data collected from a wide variety of experts and care professionals underscores the need for investment in training, communication, and preparedness to ensure a smooth transition and maximize the benefits of the new classification system.

**Disclosure of Interest:**

None Declared

